# Flow cytometric analysis of the SARS coronavirus 2 antibodies in human plasma

**DOI:** 10.1038/s41598-025-92389-8

**Published:** 2025-03-25

**Authors:** Jia-Long Fang, Leeza Shrestha, Frederick A. Beland

**Affiliations:** Division of Biochemical Toxicology, National Center for Toxicological Research, U.S. Food and Drug Administration, Jefferson, AR 72079 USA

**Keywords:** Anti-SARS-CoV-2 antibody, COVID-19, Spike protein subunit, Receptor binding domain, Nucleocapsid protein, Immunology, Medical research

## Abstract

COVID-19 is an infectious disease caused by the severe acute respiratory syndrome coronavirus (SARS-CoV-2). Anti-SARS-CoV-2 antibodies can provide information on patient immunity, identify asymptomatic patients, and track the spread of COVID-19. Efforts have been made to develop methods to detect anti-SARS-CoV-2 antibodies in humans. Here, we describe a flow cytometric assay for the simultaneous detection of anti-SARS-CoV-2 IgG and IgM in human plasma. To assess the antibody response against the different SARS-CoV-2 structural proteins, five viral recombinant proteins, including spike protein subunit 1 (S1), N-terminal domain of S1 (S1A), spike receptor-binding domain (RBD), spike protein subunit 2 (S2), and nucleocapsid protein (N), were generated. A comparison of the antibody profiles detected by the assay with plasma from 100 healthy blood donors collected prior to the COVID-19 pandemic and plasma from 100 virologically confirmed COVID-19 patients demonstrated a clear discrimination between the two groups. Among the COVID-19 patients, the antibody responses for the viral proteins, as determined by their prevalence, were anti-RBD IgG = anti-N IgG > anti-S1 IgG > anti-S1A IgG > anti-S2 IgG, and anti-RBD IgM > anti-S1 IgM > anti-N IgM > anti-S2 IgM. The prevalence of anti-SARS-CoV-2 IgG and IgM was not associated with sex, age, race, days after the onset of symptoms, or severity of illness, except for a higher prevalence of anti-S2 IgG being observed in men than in women. The levels of anti-RBD IgG were higher in patients 65 years and older and in patients who had severe symptoms. Similarly, patients who had severe symptoms exhibited higher levels of anti-S1 and anti-S1A IgG than patients who had mild or moderate symptoms. The levels of anti-RBD IgM tended to be higher in men but did not differ among age, race, days after the onset of symptoms, or severity of illness. Our study indicates that the flow cytometric assay, especially using RBD as target antigen, can be used to detect simultaneously anti-SARS-CoV-2 IgG and IgM antibodies in human plasma.

## Introduction

Human coronaviruses were first identified in the mid-1960s and are a large group of enveloped positive-sense RNA viruses. Of the seven coronaviruses that infect humans, SARS-CoV, MERS-CoV, and SARS-CoV-2 cause severe respiratory disease, with SARS-CoV-2 leading to a much higher infection rate than SARS-CoV or MERS-CoV. SARS-CoV-2 was discovered in December 2019, has since spread globally, and caused an outbreak of respiratory tract disease known as Coronavirus Disease 2019 (COVID-19). The viral genome of SARS-CoV-2 is approximately 30 kb in length and encodes four structural proteins (spike (S), envelope, membrane, and nucleocapsid (N)) that form the structurally complete viral particle, multiple non-structural proteins, and other accessory proteins^1–3^. SARS-CoV-2 infects host cells through its S protein, which is the most important surface protein and has two subunits, S1 and S2, where S1 is for host cell receptor binding and S2 is for membrane fusion. S1 is further comprised of an N-terminal domain (S1A), receptor binding domain (RBD), subdomain C, and subdomain D^3,4^. Once the virus enters the cells, the host immune system recognizes the whole virus or its surface epitopes, eliciting innate and/or adaptive immune responses^5^. The humoral immune response, including the production of antibodies against SARS-CoV-2, is essential to control and fight the viral infection.

The N protein is the most abundantly expressed protein during SARS-CoV-2 infection^6^. The S and N proteins are the most immunogenic and are used as antigens in many methods for diagnosing SARS-CoV-2 infection^7^. Only antibodies against the S protein, specifically the RBD, can induce protective immunity against SARS-CoV-2 infection^8–10^. Thus, S, RBD, or N are the primary proteins used as target antigens for SARS-CoV-2 antibody tests. Since the S protein is being used as a leading target antigen in vaccine development, the detection of anti-S and anti-N specific antibodies may indicate previous infection and/or vaccination^11–13^.

Although real-time PCR for viral RNA detection remains the gold standard for diagnosing SARS-CoV-2 infection^14^, anti-SARS-CoV-2 antibodies are crucial for understanding patient immunity, identifying asymptomatic carriers, and determining the true prevalence of COVID-19^15^. Unlike real-time PCR, anti-SARS-CoV-2 antibodies can be detected throughout the course of infection^14,16^. Several techniques, including lateral flow immunoassays^17^, ELISA^7^, cell-based flow cytometric assays^18–21^, and bead-based multiplex flow cytometric assays^11,22^, have been employed to detect anti-SARS-CoV-2 antibodies in serum, plasma, and whole blood. Generally, flow cytometric methods offer higher specificity and sensitivity compared to ELISAs or lateral flow immunoassays, and provide an alternative approach for analyzing anti-SARS-CoV-2 antibodies. However, existing cell-based flow cytometric assays face challenges for high-throughput screening, as they require advanced planning to ensure an adequate supply of cells. Bead-based multiplex flow cytometry, while offering higher sensitivity and multiplexing capabilities for measuring antibodies against multiple viral antigens simultaneously, is limited by the high cost of the required flow cytometer.

**Fig. 1:**
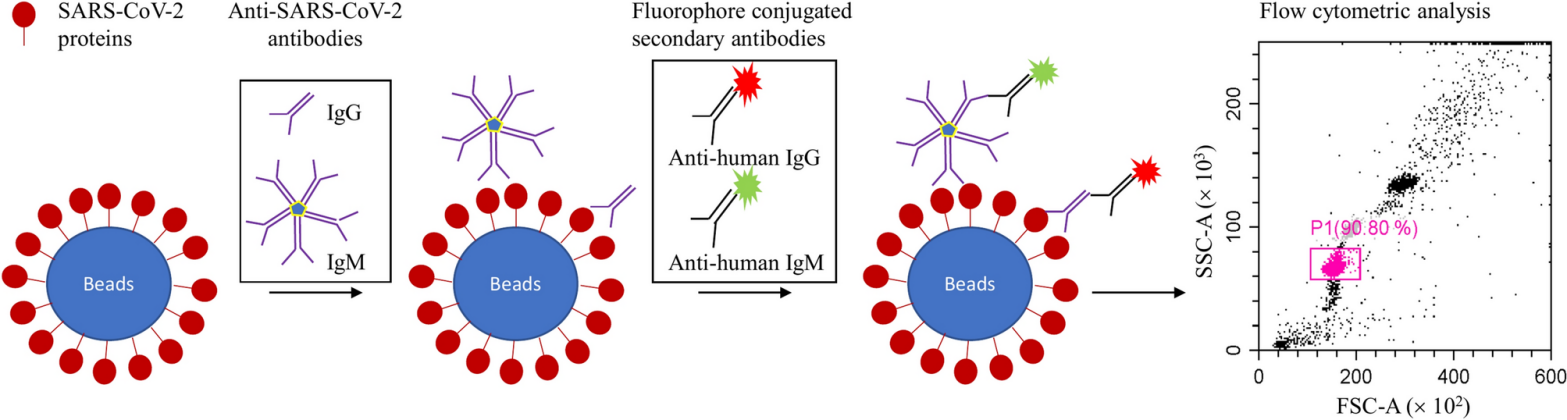
Schematic representation of the flow cytometric assay.

In the present study, we developed a bead-based flow cytometric assay to detect simultaneously SARS-CoV-2 IgG and IgM antibodies in human plasma samples (Fig. 1). To evaluate the antibody response against different structural proteins of the SARS-CoV-2 Wuhan strain, five viral recombinant proteins were generated: S1(Val16 – Arg682), S1A (Val16 – Asp294), RBD (Phe329 – Cys538), S2 (Ser686 – Pro1213), and N (Met1 – Ala419), were generated. This flow cytometric assay is reliable, highly accurate, and suitable for implementation in various medical and biological laboratories. The assay effectively detects anti-SARS-CoV-2 antibodies to aid in immunity assessment, identify asymptomatic carriers, and determine COVID-19 prevalence.

## Materials and methods

### Plasma samples

Plasma samples of healthy blood donors (n = 100) and COVID-19 patients (n = 100) were purchased from BioIVT (Hicksville, NY). The characteristics of the donors and COVID-19 patients are listed in Table 1. The plasma samples from healthy blood donors (negative panel) were collected prior to the outbreak of COVID-19, with ages ranging from 21 to 77 years (median age 48 years). Among the negative panel, 59 donors were female, and 41 donors were male. COVID-19 patients were diagnosed and confirmed using a standard real-time PCR assay for SARS-CoV-2 infection. The plasma samples from COVID-19 patients were collected before October 2020, which was before COVID-19 vaccines were available. The ages of the COVID-19 patients ranged from 21 to 76 years, with a median age of 47 years. Among them, 59 patients were female, and 41 patients were male. The presence of anti-SARS-CoV-2 IgG and IgM in plasma samples from the COVID-19 patients was examined by at least one of the following serology tests: Diazyme-IgG/IgM, Viracor-IgG/IgM, Epitope-EDI-IgG/IgM, or Architect-IgG. The number of days after the onset of symptoms and clinical classifications of severity for COVID-19 patients were obtained from the clinical records. Plasma samples were stored at -80 °C until analysis.

**Table 1:** Characteristics (sex, age, and race) of healthy blood donors and COVID-19 patients.

	Healthy blood donors	COVID-19 patients
Sex		
Female	59	59
Male	41	41
Age (years)		
≤ 24	5	6
25—64	90	86
≥ 65	5	8
Race		
Caucasian	10	19
Hispanic	66	67
Black	20	3
Asian	1	3
Other	3	4
Unknown	0	4

### Materials

CML microsphere beads (4% w/v, 2 µm), aldehyde/sulfate microsphere beads (4% w/v, 2 µm), FreeStyle 293 expression medium, and FreeStyle 293F cells were purchased from Invitrogen (Carlsbad, CA). Human anti-N and anti-S1 IgG and IgM were obtained from GenScript (Piscataway, NJ). Human anti-RBD IgG and IgM were acquired from InvivoGen (San Diego, CA). Human anti-S2 IgG was purchased from Cell Sciences (Newburyport, MA). Rabbit anti-Histag IgG was purchased from Cell Signaling Technology (Danvers, MA). Alexa Fluor 647 AffiniPure donkey anti-human IgG and Alexa Fluor 488 AffiniPure donkey anti-human IgM were acquired from Jackson ImmunoResearch Laboratories Inc (West Grove, PA). Bovine serum albumin fraction V (BSA) was purchased from Sigma-Aldrich, Inc. (St. Louis, MO).

### Cell culture

FreeStyle 293F cells were cultured in FreeStyle 293 expression medium supplemented with penicillin–streptomycin solution at 37 °C in a humidified atmosphere with 8% CO_2_ on an orbital shaker platform rotating at 125 rpm.

### Production of recombinant SARS-CoV-2 structural proteins

The coding sequences of the structural proteins of the SARS-CoV-2 Wuhan strain (Table 2) were inserted into the pcDNA3.4 mammalian expression vector, resulting in the construct pcDNA3.4-signal peptide-SARS-CoV2-subunit-8xHis (Biosettia, San Diego, CA). FreeStyle 293F cells were then transfected with the generated SARS-CoV-2 subunit expression vectors using a cationic lipid-based transfection reagent 293fectin. Briefly, 3 × 10^7^ cells, with viability ≥ 98%, were seeded in Nalgene single-use PETG Erlenmeyer flasks (125 ml) containing 28 mL of FreeStyle 293F expression medium. Aliquots (30 μg) of the pcDNA3.4 expression vector and 60 μl of 293fectin were each diluted in 1 mL of Opti-MEM I, incubated for 5 min at room temperature, and then the diluted vector was added to the diluted 293fectin for a 30 min incubation at room temperature. Finally, the 2 mL mixture was added to the 28 mL of FreeStyle 293F cells. Two to five days post-transfection, the cells were harvested and centrifuged at 800 rpm for 5 min at room temperature. The supernatants, which contained the recombinant SARS-CoV-2 proteins fused with 8xhis-tag at the C-terminus, were collected and stored at 4 °C until protein purification.

**Table 2:** GenBank accession numbers, protein ID, and amino-acid residues of each SARS-CoV-2 subunits.

Proteins	Accession #	Protein ID	Amino-acid residues
Nucleocapsid protein (N)	MN908947.3	QHD43423.2	Met1 – Ala419
Spike protein subunit 1 (S1)		QHD43416.1	Val16 – Arg682
Spike protein subunit 2 (S2)			Ser686 – Pro1213
Receptor binding domain (RBD)			Phe329 – Cys538
N-terminal domain of S1 (S1A)			Val16 – Asp294

### Purification and identification of recombinant SARS-CoV-2 structural proteins

**Fig. 2:**
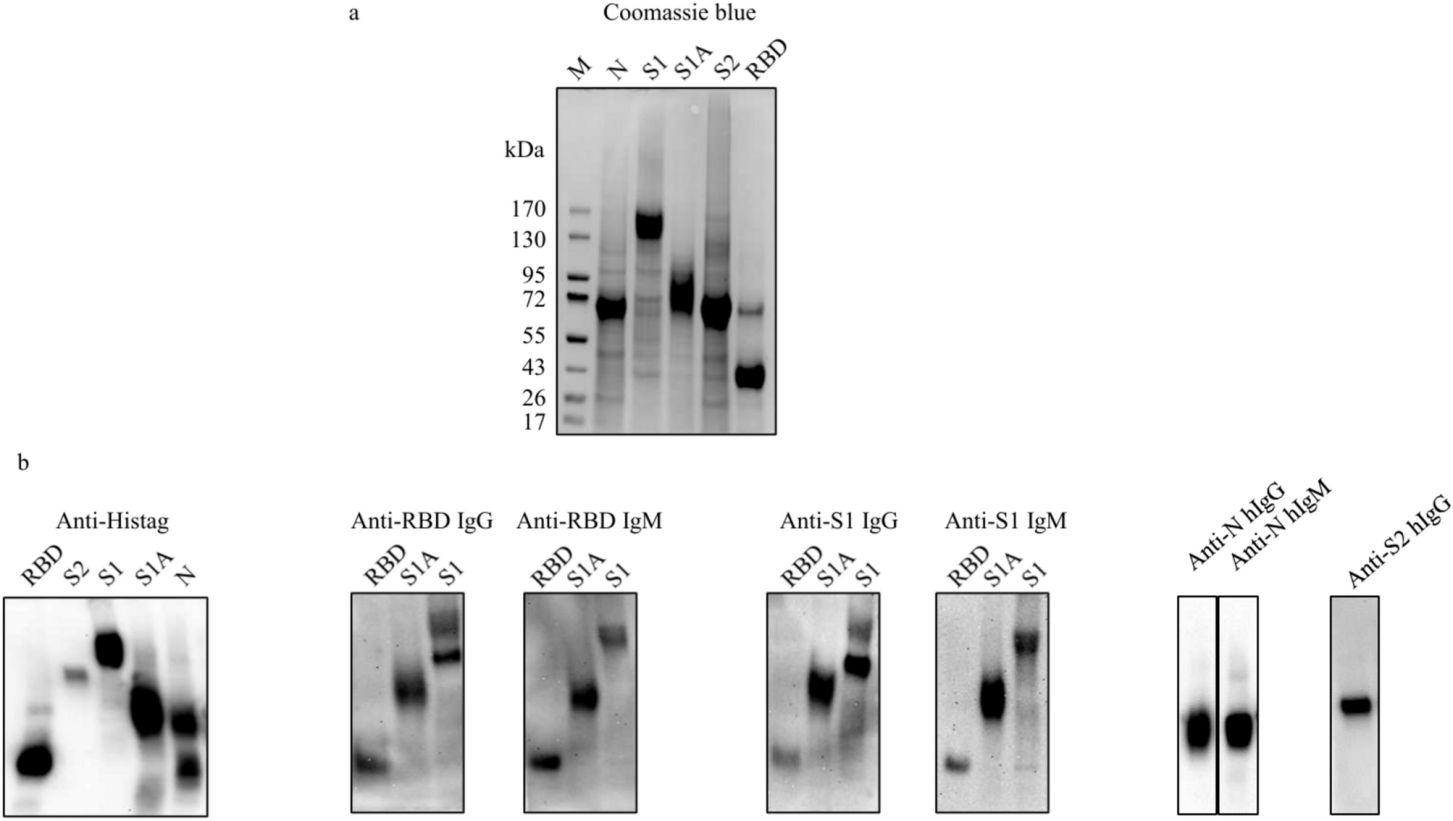
Coomassie blue staining (**a**) and Western blotting of the purified recombinant SARS-CoV-2 proteins (**b**). All the human anti-SARS-CoV-2 protein positive antibodies (IgG and/or IgM) were diluted at 1:1,000; the dilution for rabbit anti-Histag IgG was 1:2,000. M: protein molecular marker.

All the recombinant SARS-CoV-2 structural proteins were purified by PureCube 100 INDIGO Ni-Agarose (Cube Biotech, Wayne, PA) following the manufacturer’s manual. Each eluate was collected and desalted using an Amicon Ultra-15 10 K centrifugal filter device (Thermo Fisher Scientific, Inc., Pittsburgh, PA). The protein concentrations of the recombinant SARS-CoV-2 proteins were determined using a BCA protein assay (Thermo Fisher Scientific, Inc.). The purity of the purified recombinant SARS-CoV-2 proteins was > 90% as analyzed by SDS-PAGE using 6% Bis–Tris gels with Coomassie blue staining (Fig. 2a). The bands corresponding to the recombinant SARS-CoV-2 proteins were cut and the protein identification of each band was determined by MALDI TOF/TOF tandem mass spectrometric analysis (Applied Biomics, Hayward, CA). The protein identification analysis demonstrated a protein score confidence interval % at 100, indicating all the recombinant proteins (S1A, S1, RBD, S2, and N) were correct.

### Western blot analysis

The purified recombinant SARS-CoV-2 proteins were further examined by Western blot analysis. Five micrograms of the purified recombinant SARS-CoV-2 proteins were loaded into 6% Bis–Tris gels, separated by gel electrophoresis, and transferred onto a polyvinylidene difluoride membrane. Membranes were blocked with 5% milk in phosphate-buffered saline (PBS) and then incubated with the appropriate commercially available human anti-SARS-CoV-2 protein antibodies followed by a specific horseradish peroxidase-conjugated anti-human IgG (1:30,000) or IgM (1:15,000), or anti-rabbit IgG (1:20,000) secondary antibody. The blots were detected by chemiluminescence using Immobilon Western Horseradish Peroxidase Substrate (Millipore Corporation, Billerica, MA), a FluorChem R System (ProteinSimple, San Jose, CA), and Digital Darkroom software for acquisition and analysis (ProteinSimple).

### Flow cytometric assay

The SARS-CoV-2 structural protein conjugated beads (target beads) were either the N protein bound to Aldehyde/Sulfate beads or S1, S1A, RBD, or S2 protein coupled to CML beads. Aldehyde/Sulfate beads were used with the N protein because the N protein exhibited weak binding to the CML beads. In addition, two control beads (5% BSA coupled CML beads or Aldehyde/Sulfate beads) were also prepared. The control beads compensate for nonspecific binding of human plasma to the beads, which could potentially lead to false positives. The same batch of BSA was used throughout the study to account for batch-to-batch variation. The coupling procedures were conducted according to the manufacturer’s recommendations. Briefly, both Aldehyde/Sulfate and CML beads were equilibrated with 50 mM 2-(*N*-morpholino)ethanesulfonic acid activation buffer, pH 6.0; the final beads suspension was at approximately 20 mg/mL (2% w/v). SARS-CoV-2 proteins or BSA were also prepared at 1 mg/mL in 50 mM 2-(*N*-morpholino)ethanesulfonic acid activation buffer. To activate CML beads, aliquots (5 mL) of CML beads and 200 mg of 1-(3-dimethylaminopropyl)-3-ethylcarbodiimide were mixed at room temperature for 30 min. The activated CML beads were then centrifuged and washed 2 times with 50 mM 2-(*N*-morpholino)ethanesulfonic acid activation buffer to give a final bead suspension of approximately 20 mg/mL (2% w/v). Aliquots (5.0 mg) of SARS-CoV-2 proteins or BSA were incubated with either Aldehyde/Sulfate beads (100 mg) or the activated CML beads (100 mg) at room temperature for 4 h with gentle mixing. The mixture was centrifuged at 2,500 g for 20 min at room temperature to separate protein conjugated beads from unbound protein and the conjugated beads were resuspended and washed 3 times with PBS. All beads were resuspended in 10 mL of 0.1 M PBS, pH 7.2, 0.5% BSA, 0.02% NaN_3_, giving a final concentration of 1% solids, and stored at 4 °C.

To analyze anti-SARS-CoV-2 antibodies in human plasma samples, 35 µl of freshly diluted target beads or control beads (1 to 100 dilution using 0.5% BSA in PBS) were incubated with diluted human plasma (2.5 µl plasma and 47.5 µl 5% BSA in PBS) and 1 mL 5% BSA in PBS using an Eppendorf Thermomixer R (Eppendorf North America, Hauppauge, NY) at 37 ºC, with shaking at 800 rpm, for 30 min. The beads were then washed 3 times with 5% BSA in PBS, resuspended in 500 µl 5% BSA in PBS, and stained for bound IgG and IgM with a mixture of a specific Alexa Fluor 647-conjugated anti-human IgG and a specific Alexa Fluor 488-conjugated anti-human IgM secondary antibody. After incubation at 37 ºC with shaking at 800 rpm for 30 min, the beads were washed 3 times with 5% BSA in PBS and analyzed on a Beckman Coulter CytoFlex flow cytometer (Indianapolis, IN). Data were acquired and analyzed using CytExpert 1.2 Software (Beckman Coulter). The forward-scatter (FSC) signal and the side-scatter (SSC) signal were measured in the linear mode, with a gain of 20 for FSC and 30 for SSC. Fluorescence was detected on a logarithmic scale. A total of 20,000 single beads was analyzed for each sample. The difference in median fluorescent intensity (MFI) between the target beads and the control beads was used to determine anti-SARS-CoV-2 antibody positivity and the levels of anti-SARS-CoV-2 antibodies. Commercially available human anti-SARS-CoV-2 RBD, S1, N, and S2 antibodies were used as positive anti-SARS-CoV-2 antibody standards and 5% BSA was used as a blank control. The fluorescein conjugated anti-human IgG and IgM secondary antibodies were affinity-purified and further tested by flow cytometry to ensure no cross-reaction with other human immunoglobulins. The reactivity of both target and control beads with the anti-his-tag antibody was evaluated using the flow cytometric assay, and no response was observed with the antibody.

### Statistical analyses

Statistical analyses were performed using GraphPad Prism 6 (GraphPad Software, LLC, San Diego, CA). Normality of data was analyzed using the Kolmogorov–Smirnov test. Variations in the prevalence of anti-SARS-CoV-2 IgG or IgM with respect to sex, age, race, days after the onset of symptoms, or severity of illness were assessed using Chi-square analysis. The Kruskal–Wallis with Dunn’s multiple comparison test was used for comparison of the level of anti-SARS-CoV-2 IgG or IgM among multiple groups in terms of sex, age, race, days after the onset of symptoms, or severity of illness. All tests were two-tailed, and a *p* < 0.05 was considered statistically significant.

## Results

### Flow cytometric assay development

Western blotting of the purified recombinant SARS-CoV-2 structural proteins reveal that all the recombinant proteins were his-tagged and readily detected using positive anti-SARS-CoV-2 structural protein IgG and IgM (Fig. 2b).

**Fig. 3:**
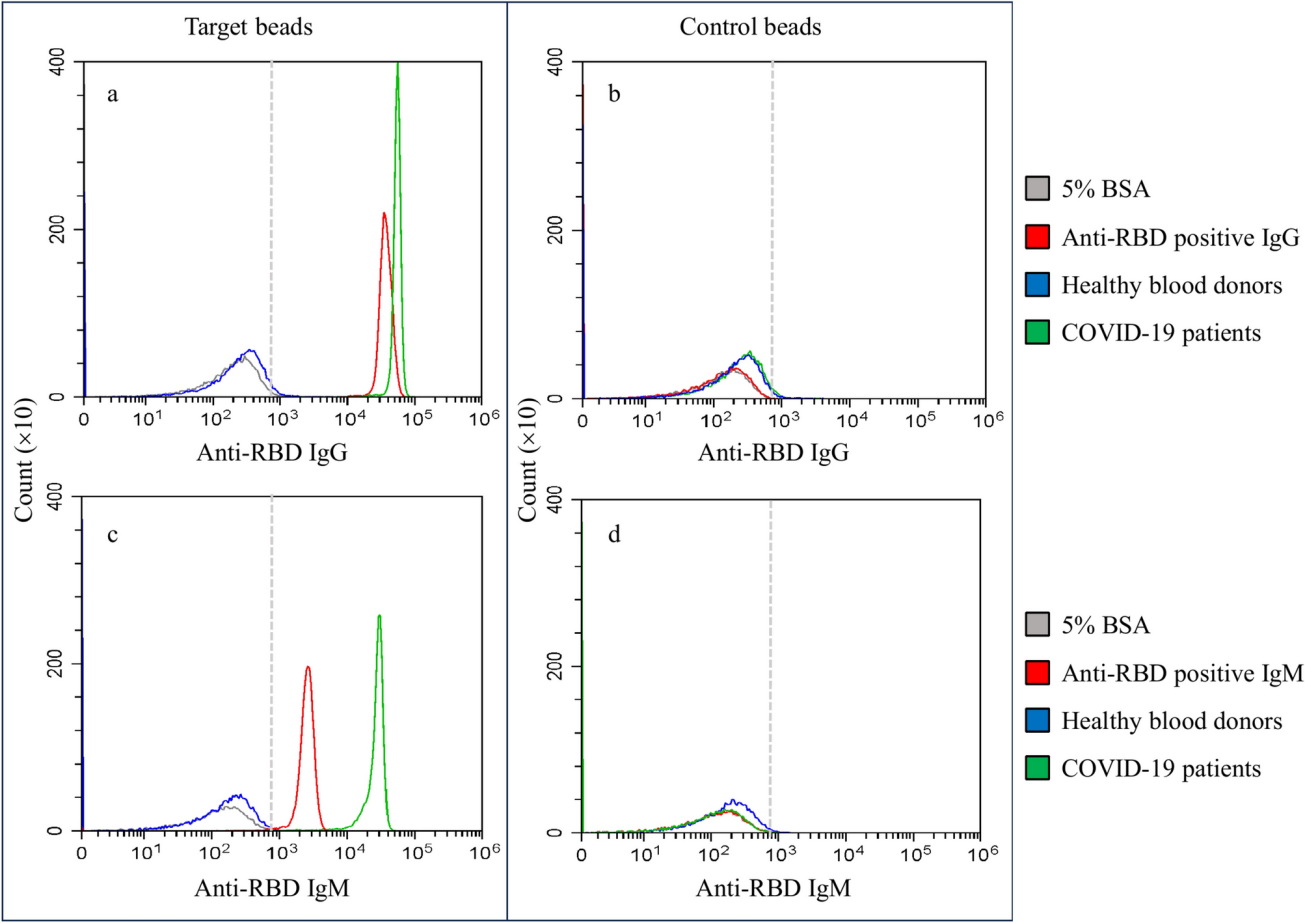
Representative flow cytometry histograms showing the detection of anti-RBD IgG (**a**, **b**) and IgM (**c**, **d**) in plasma samples of healthy blood donors and COVID-19 patients. RBD conjugated target beads (**a**, **c**) or the control beads (**b**, **d**) were incubated with negative control (5% BSA, grey), anti-RBD IgG or IgM positive standard (red), and human plasma samples (healthy blood donor, blue; COVID-19 patient, green) as described in the Materials and Methods. The dashed line indicates the cutoff-point for the assay based on fluorescent intensity.

After confirmation of the recombinant SARS-CoV-2 proteins, viral protein conjugated target beads and 5% BSA coupled control beads were prepared. Using the beads, a flow cytometric assay was developed to detect simultaneously anti-SARS-CoV-2 IgG and IgM in human plasmas. The histograms for all 5 target antigens (S1, S1A, RBD, S2, and N) conjugated target beads and the control beads were similar. A representative histogram obtained with RBD-conjugated target beads is shown in Fig. 3 and clearly demonstrates higher fluorescent intensity in the plasma sample from a COVID-19 patient than both the healthy blood donor and blank control (Fig. 3a, c). With the control beads, fluorescent intensity in the plasma sample from the COVID-19 patient and the commercial anti-RBD IgG or IgM was close to the blank control and the plasma from the healthy blood donor (Fig. 3b, d).

A cut-off point for assay positivity was determined using plasmas collected from the negative panel. The plasma samples were tested in at least 2 separate experiments with the viral protein-conjugated target beads and the control beads. After complete analysis of the negative panel, the cut-off point was defined to be the average MFI of the negative panel plus three standard deviations. Plasma was considered positive for anti-SARS-CoV-2 IgG or IgM if its MFI was greater than the cut-off point value in at least 2 separate experiments.

### Anti-SARS-CoV-2 antibodies in human plasma

**Fig. 4:**
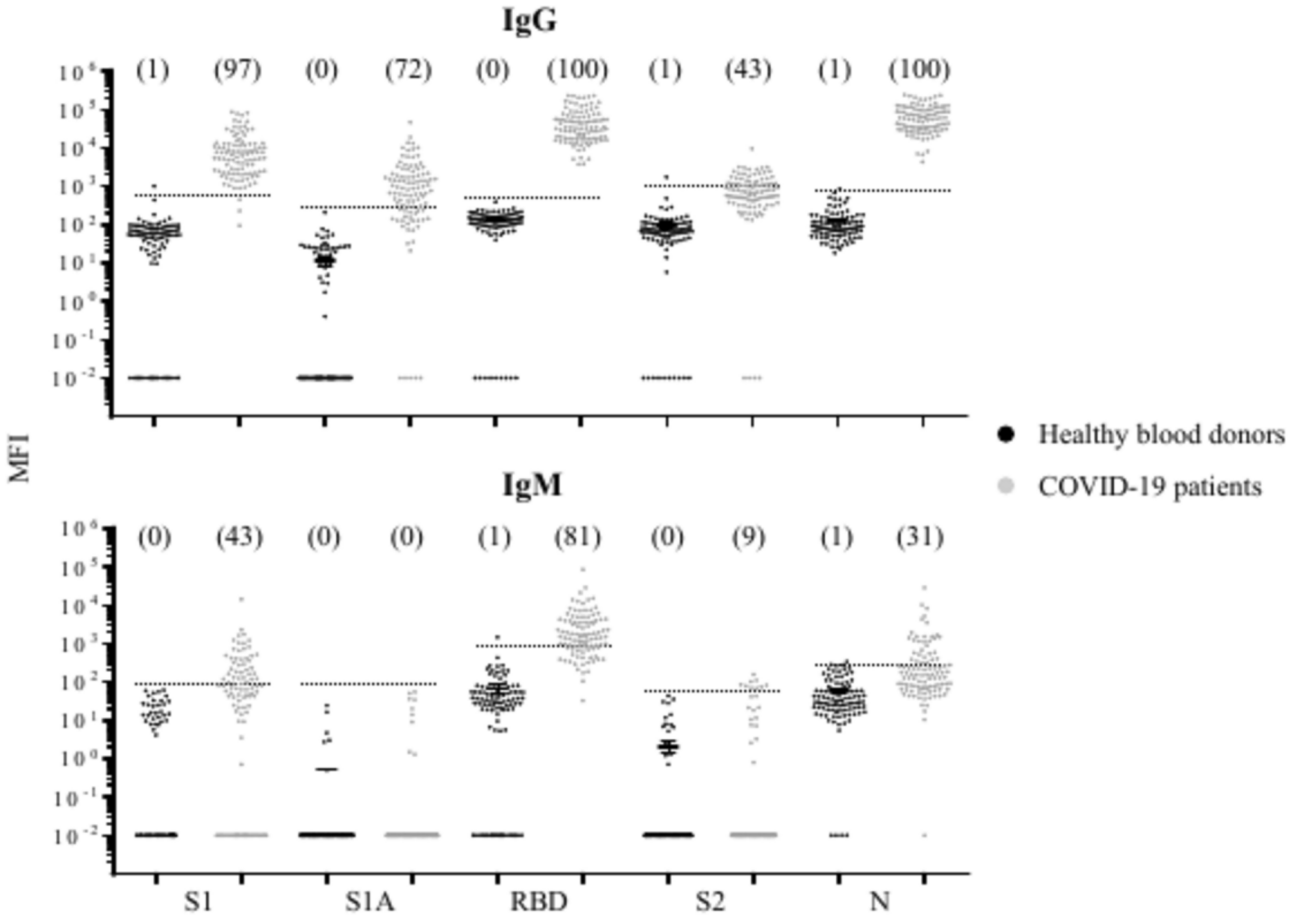
Flow cytometric analysis of SARS-CoV-2 antibodies against target antigens in plasma samples from healthy blood donors (n = 100) collected before the COVID-19 pandemic and from real-time PCR confirmed COVID-19 patients (n = 100). MFI values were obtained by subtracting the MFI values of the control beads from the MFI values of the target beads. The dashed line indicates the cutoff-point for the assay. The number in parathesis indicate the number of samples with an anti-SARS-CoV-2 antibody response above the cutoff-point.

Antibody reactivity to the 5 target antigens was evaluated for anti-SARS-CoV-2 IgG and IgM in plasmas from 100 healthy blood donors collected prior to COVID-19 outbreak and plasma samples from 100 real-time PCR-positive COVID-19 patients. As shown in Fig. 4 and Supplementary Table 1, in healthy blood donors’ group, only a single sample, at the low end of the assay dynamic range, was detected as positive for each antigen; thus, the specificity (true negative rate) of detection was 99 or 100%, depending upon specific antibody. In the COVID-19 patients’ group, IgG was more reactive than IgM against each of the SARS-CoV-2 antigens. Anti-RBD-IgG and anti-N-IgG showed a sensitivity (true positive rate) of detection of 100%, followed by anti-S1-IgG (97%), anti-S1A-IgG (72%), and anti-S2-IgG (43%). The positive prediction and negative prediction values with anti-RBD-IgG were 100 and 100%; slightly lower values were observed with anti-N-IgG (99 and 100%) and anti-S1-IgG (99 and 97%). A lower specificity of detection was observed with IgM: anti-RBD-IgM gave a value of 81%, followed by anti-S1-IgM (43%), anti-N-IgM (31%), anti-S2-IgM (9%), and anti-S1A-IgM (0%). The antibody responses for the viral proteins as determined by the prevalence were anti-RBD IgG = anti**-**N IgG > anti**-**S1 IgG > anti**-**S1A IgG > anti**-**S2 IgG, and anti**-**RBD IgM > anti**-**S1 IgM > anti**-**N IgM > anti**-**S2 IgM.

### Correlation of sex, age, race, days after the onset of symptoms, or severity of illness with anti-SARS-CoV-2 antibodies in COVID-19 patients

**Fig. 5:**
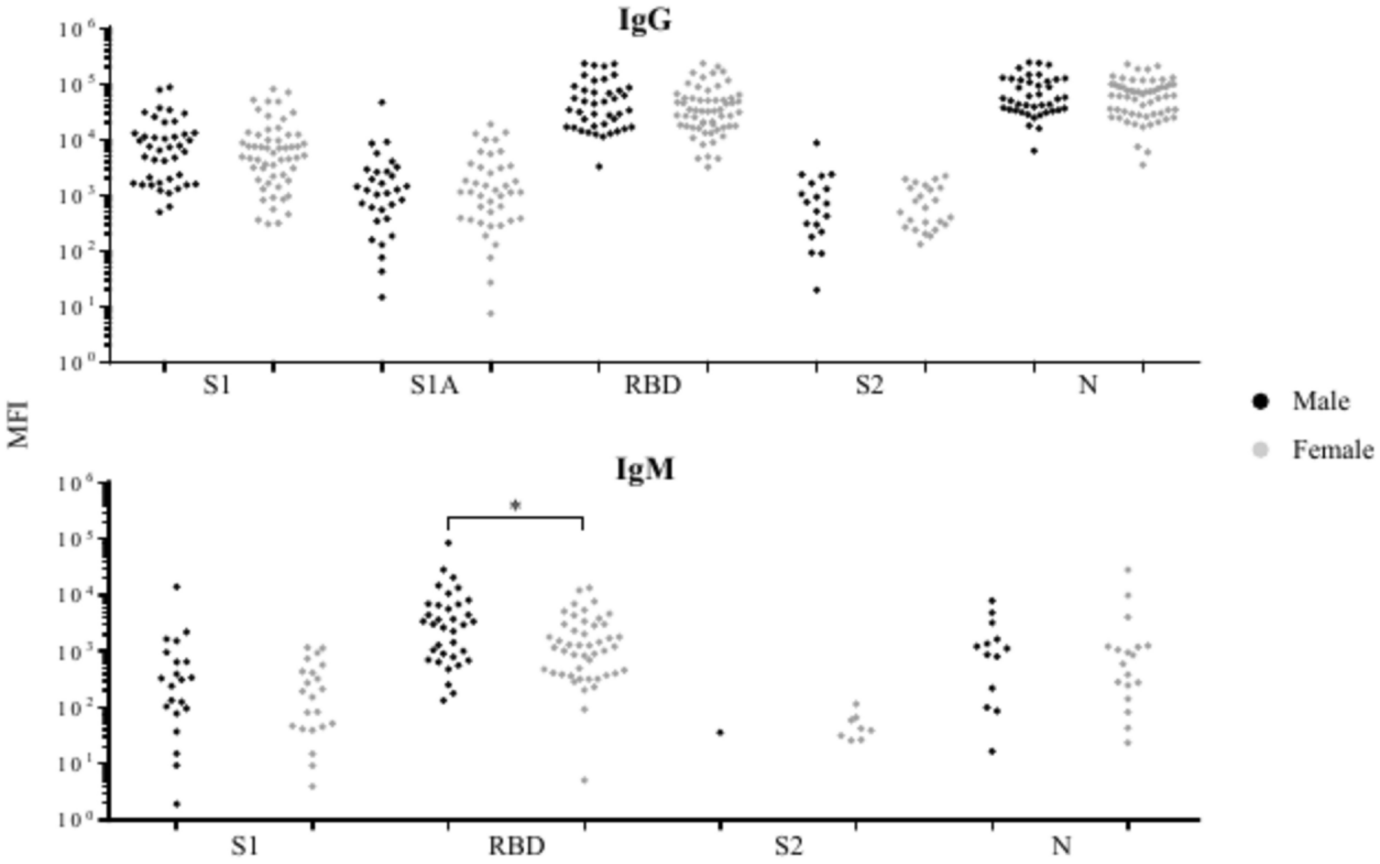
Comparisons of the levels of SARS-CoV-2 antibodies against target antigens in male and female COVID-19 patients who are positive (MFI above cutoff-off point) for anti-SARS-CoV-2 IgG or IgM. The number of samples with a positive anti-SARS-CoV-2 antibody response is reported in the Supplementary Table 2. *Significant (p < 0.05) difference between males and females.

The relationship between the prevalence or levels of anti-SARS-CoV-2 IgG or IgM in COVID-19 patients and their demographic characteristics, including sex, age, race, days after the onset of symptoms, or severity of symptoms was analyzed. The prevalence of anti-S2 IgG was significantly lower in female patients (32.2%) as compared to male patients (58.5%) (Supplementary Table 2). This was not due to differences in days after disease onset, which did not differ between the sexes. The levels of anti-SARS-CoV-2 IgG and IgM against all target antigens ranged widely in both men and women. The level of anti-RBD IgM in positive plasma samples was significantly higher in men compared to women (Fig. 5). None of the other comparisons between men and women was statistically significant.

**Fig. 6:**
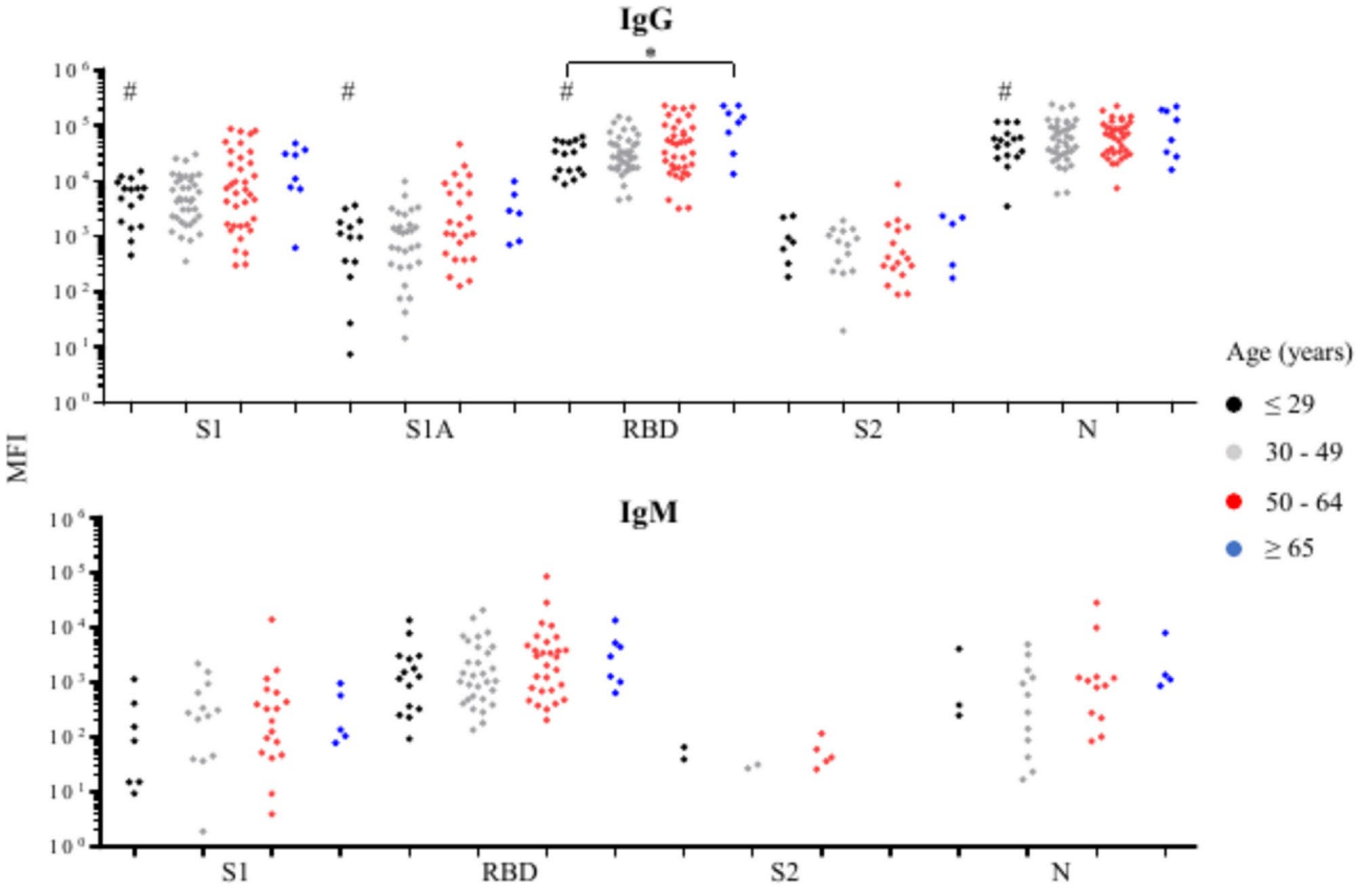
Comparisons of the levels of SARS-CoV-2 antibodies against target antigens in age groups among COVID-19 patients who are positive (MFI above cutoff-point) for anti-SARS-CoV-2 IgG or IgM. The number of samples with a positive anti-SARS-CoV-2 antibody response is reported in the Supplementary Table 3. #Significant (p < 0.05) age-related trend. *Significant (p < 0.05) difference between the indicated age groups.

The plasma samples were separated into 4 age groups (≤ 29, 30 – 49, 50—64 and ≥ 65 years) based on the classification of age groups by Centers for Disease Control and Prevention (CDC) Data tracker -Antibody Seroprevalence (CDC COVID Data Tracker: Antibody Seroprevalence). The prevalence of anti-SARS-CoV-2 IgG or IgM against all target antigens did not vary significantly among age groups (Supplementary Tables 3). The levels of anti-SARS-CoV-2 IgG and IgM against all target antigens ranged widely across all age groups (Fig. 6). A significant correlation was found between age and the increased levels of anti-S1 IgG, anti-S1A IgG, anti-RBD IgG, and anti-N IgG (Fig. 6). COVID-19 patients ≥ 65 years had a significantly higher level of anti-RBD IgG than patients ≤ 29 years. This was not due to differences in disease severity, which did not differ between the two age brackets. None of the other comparisons among age groups was statistically significant (Fig. 6).

**Fig. 7:**
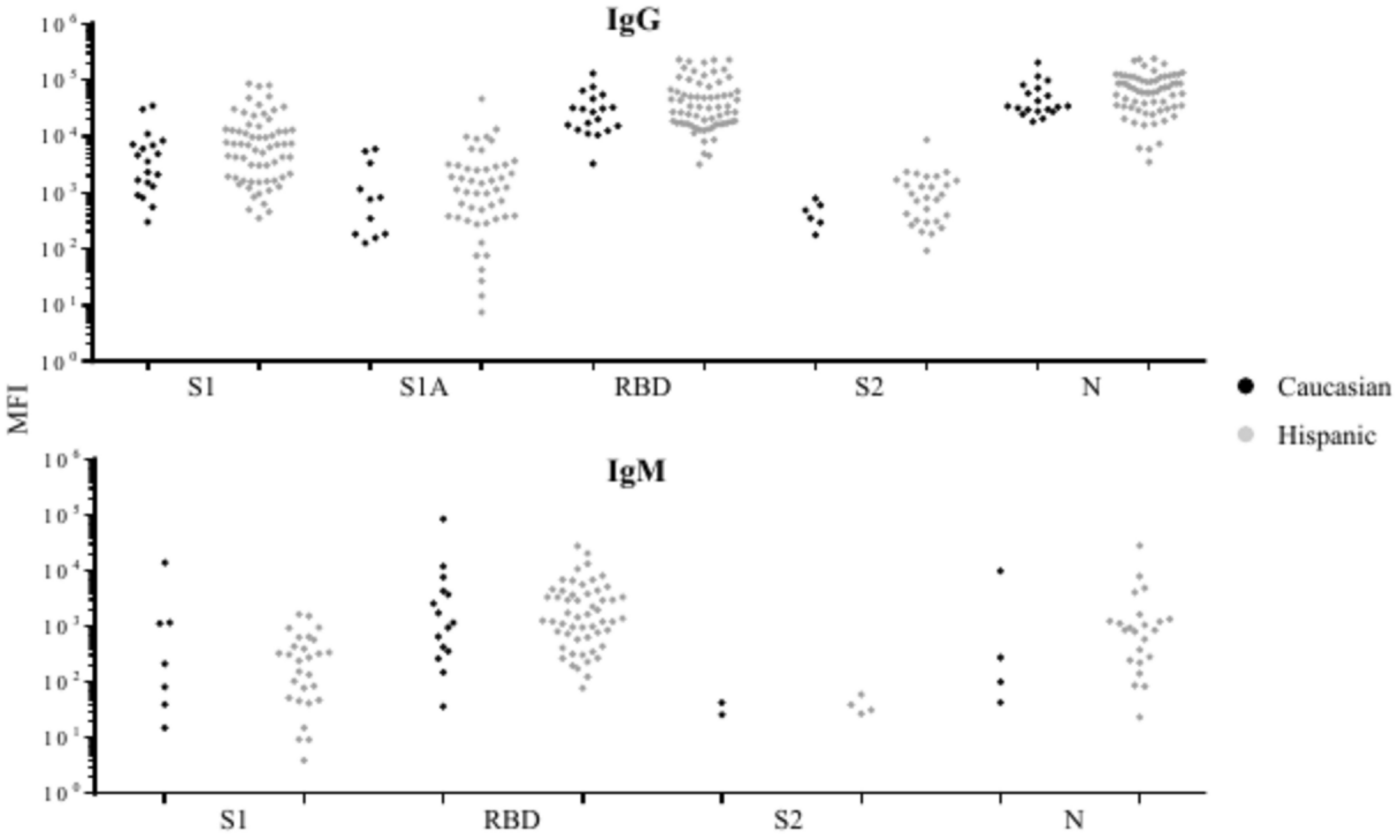
Comparisons of the levels of SARS-CoV-2 antibodies against target antigens in Caucasian and Hispanic COVID-19 patients who are positive (MFI above cutoff-point) for anti-SARS-CoV-2 IgG or IgM. The number of samples with a positive anti-SARS-CoV-2 antibody response is reported in the Supplementary Table 4.

The correlation of race with the prevalence and levels of anti-SARS-CoV-2 IgG and IgM against the target antigens in COVID-19 patients was also analyzed. The statistical analysis was restricted to Caucasians and Hispanic due to the limited number of samples in the other racial groups. The differences between Caucasian and Hispanic in the prevalence (Supplementary Table 4) or levels (Fig. 7) of anti-SARS-CoV-2 IgG or IgM against all the target antigens were not significant.

**Fig. 8:**
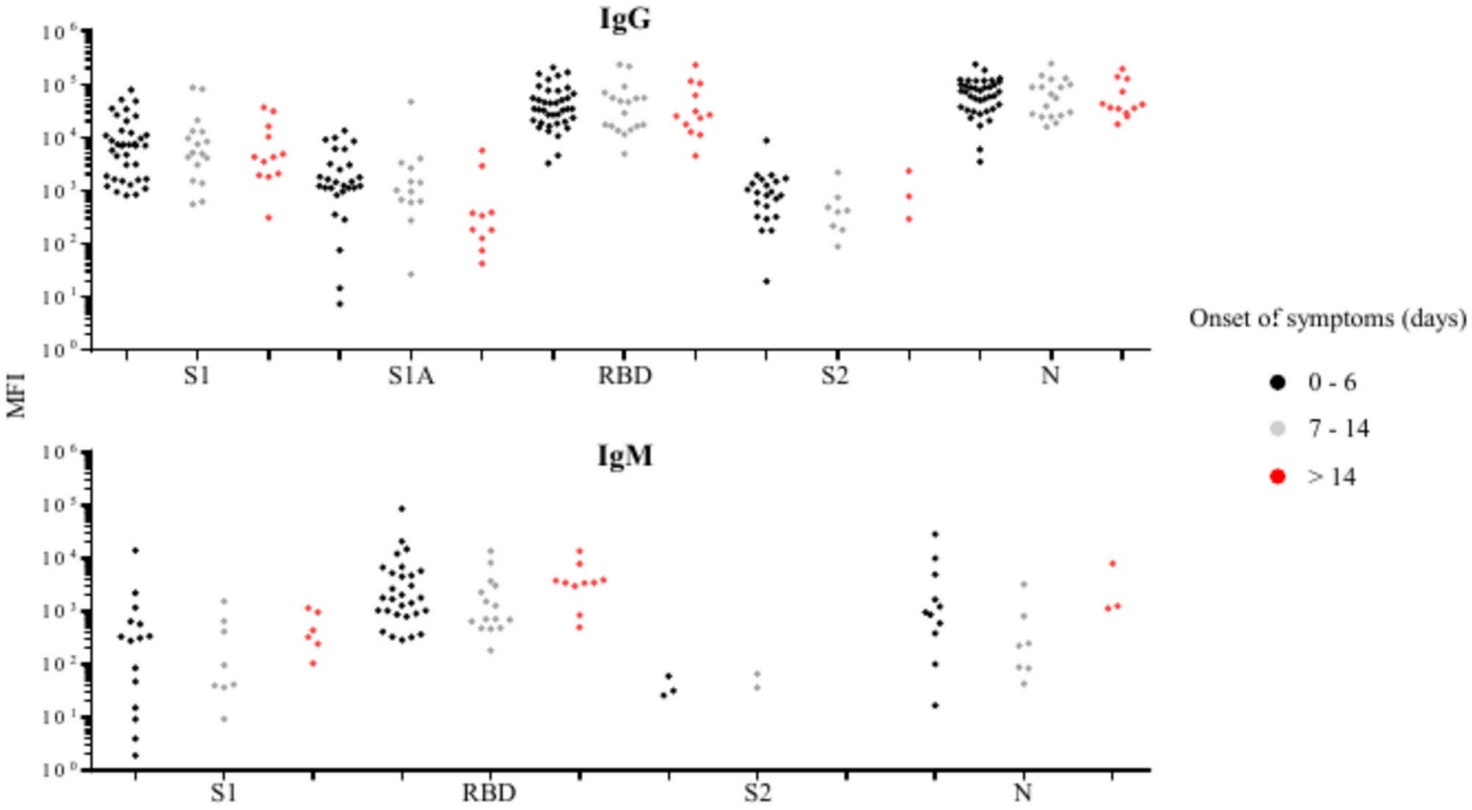
Comparisons of the levels of SARS-CoV-2 antibodies against target antigens in days after symptom onset among COVID-19 patients who are positive (MFI above cutoff-point) for anti-SARS-CoV-2 IgG or IgM. The number of samples with a positive anti-SARS-CoV-2 antibody response is reported in the Supplementary Table 5.

Based on the number of days the plasma samples were obtained after the onset of symptoms, the patients were categorized into 3 groups with 0—6 (38 patients), 7—14 (18 patients), and ≥ 14 days (12 patients). Information on symptom onset was not available for 32 patients and they were excluded from this statistical analysis. Differences among days after the onset of symptoms in the prevalence (Supplementary Table 5) or levels (Fig. 8) of anti-SARS-CoV-2 IgG or IgM against all target antigens were not significant.

**Fig. 9:**
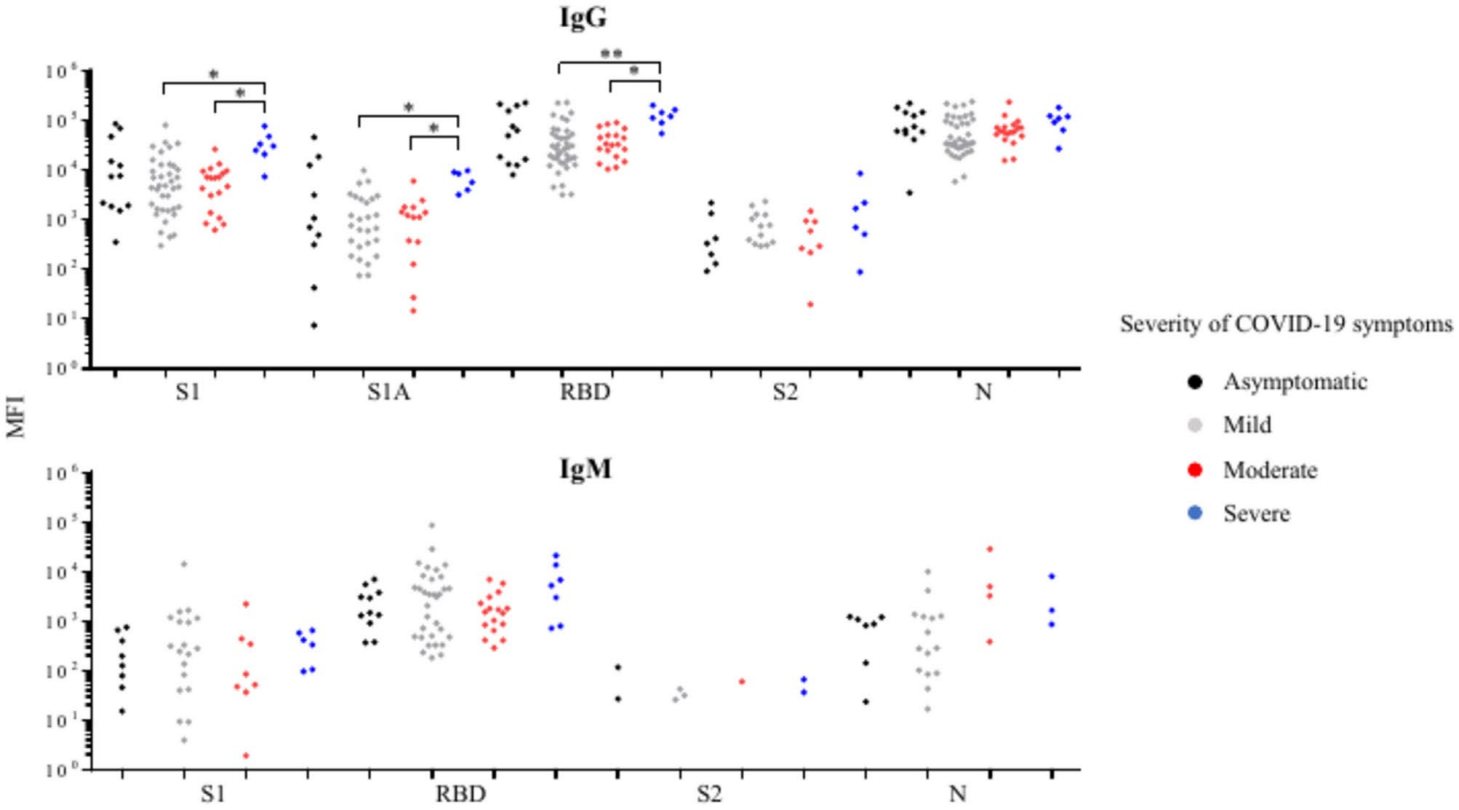
Comparisons of the levels of SARS-CoV-2 antibodies against target antigens in severity of symptoms among COVID-19 patients who are positive (MFI above cutoff-point) for anti-SARS-CoV-2 IgG or IgM. The number of samples with a positive anti-SARS-CoV-2 antibody response is reported in the Supplementary Table 6. Significant (*, p < 0.05; **, p < 0.01) difference between the indicated severities of symptoms.

The correlation of the severity of COVID-19 symptoms with the prevalence and levels of anti-SARS-CoV-2 IgG and IgM against the target antigens in the COVID-19 patients was also analyzed. Among them, there were 12, 41, 19, and 7 patients with asymptomatic, mild, moderate, and severe symptoms, respectively. The severity of symptoms was not documented for the remaining 21 patients and, thus, they were not included in this statistical analysis. The prevalence of anti-SARS-CoV-2 IgG or IgM against all target antigens did not vary significantly among COVID-19 symptoms severity groups (Supplementary Table 6). The levels of anti-S1 IgG, anti-S1A IgG, and anti-RBD IgG in COVID-19 patients with severe symptoms were significantly higher than those in patients having mild or moderate symptoms. None of the other comparisons based upon the severity of the symptoms was statistically significant (Fig. 9).

## Discussion

In this study, a flow cytometric assay was developed to detect simultaneously SARS-CoV-2 antibody IgG and IgM in human plasma samples. Out of 100 plasmas from healthy blood donors collected prior to the COVID-19 pandemic, only one sample gave a positive signal for a few of the target antigens (anti-S1-IgG, anti-S2-IgG, anti-N-IgG and IgM, anti-RBD-IgM), with the responses being at the low end of the dynamic range of the assay. Cross-reactivity with antibodies to other human coronaviruses that share genetic similarity with SARS-CoV-2 may be a factor attributing to the sporadic presence of anti-SARS-CoV-2 antibodies in the pre-pandemic plasmas^10,23,24^.

The detection of anti-SARS-CoV-2 IgG and IgM in plasma samples from COVID-19 patients by at least 1 of 4 FDA-authorized commercial serology assay kits (specific for S and/or N antigens) was 100% and 18% for anti-SARS-CoV-2 IgG and IgM, respectively, when combining data from all the four assay kits (Supplemental material- Serology test results reported on COVID-19 patients information data sheet.xlsx, which were provided by the supplier, BioIVT). Interestingly, our flow cytometric assay could detect 100%, 100%, 97%, 72%, and 43% IgG against RBD, N, S1, S1A, and S2, respectively. In comparison to anti-SARS-CoV-2 IgG, the detection of anti-SARS-CoV-2 IgM in COVID-19 patients was lower, being at 81%, 31%, 43%, and 9% IgM against RBD, N, S1, and S2, respectively. This demonstrates that our flow cytometric assay had a high sensitivity, particularly using RBD, S1, and N as the antigens.

Antigens are important factors for serological assay to detect antibodies and the antigen selected has a significant impact on the performance of the assay. Previous studies have demonstrated that the S1 protein, including RBD, of SARS-CoV-2 has very low cross-reactivity to SARS-CoV, MERS-CoV, and other 4 common human coronaviruses, whereas S2 and N show low-level cross-reactivity^10,23,24^. Our study demonstrated essentially no anti-SARS-CoV-2 antibodies in pre-pandemic human plasmas and much higher levels of anti-SARS-CoV-2 IgG and IgM in the COVID-19 patients than the commercial assay kits. Thus, the target antigens, particularly RBD, S1, and N, used in this study are highly specific and sensitive.

Being a pentamer with 10 antigen-binding domains, human IgM is characterized by a higher antigen avidity with lower antigen affinity than IgG and is usually produced against an antigen in the early stages of infection. Consistent with our observations, previous studies showed a lower prevalence of anti-SARS-CoV-2 IgM in COVID-19 patients than anti-SARS-CoV-2 IgG^25,26^, which might be due to lower assay sensitivity for IgM detection and its natural lower antigen affinity. Furthermore, several studies reported that anti-SARS-CoV-2 IgM did not usually have a higher clinical sensitivity than anti-SARS-CoV-2 IgG^14,27^, since nearly all of the IgM positive plasmas evaluated in this study were also positive for IgG. However, anti-SARS-CoV-2 IgM may play an important role in the SARS-CoV-2 antibody neutralization^28,29^.

It is not clear if the sex and age of COVID-19 patients have an impact on the prevalence or levels of anti-SARS-CoV-2 antibodies. Our study indicated male COVID-19 patients had a higher prevalence of anti-S2 IgG and levels of anti-RBD IgM than female COVID-19 patients. These results are inconsistent with previous studies that found no discernible difference in the prevalence or levels of SARS-CoV-2 IgG/IgM between men and women^30–32^. Our study also indicated that COVID-19 patients ≥ 65 years had a significantly higher level of anti-RBD IgG than patients ≤ 29 years. This finding agrees with several studies that have reported a higher level of anti-SARS-CoV-2 IgG in older COVID-19 patients^25,30,32,33^. In contrast, Young et al. reported that there were no significant differences in anti-SARS-CoV-2 IgG, IgM, or IgA against S1 based on age^34^. These discrepancies might be due to differences in assay performance of the antibody detection methods, differences in the specific antigens, or other unknown factors.

Our study shows that anti-SARS-CoV-2 IgG and IgM could be detected from the initial day (Day 0) of the onset of COVID-19 symptoms. Previous studies also reported that anti-SARS-CoV-2 IgG and IgM occur typically within 3 weeks, but as early as 2—4 days after the onset of symptoms^27,35,36^. It should be noted that the exact infection date is not clearly defined in these studies. Our study found no association between the levels of anti-SARS-CoV-2 antibodies and the number of days after the onset of symptoms, which is not consistent with other studies^32,37^. This discrepancy may be due to the nature of the other two studies, which investigated the dynamic changes of anti-SARS-CoV-2 IgG and IgM in COVID-19 patients from the time of the onset of symptoms until 210 days^32,37^.

Our research found the levels of anti-S1 IgG, anti-S1A IgG, and anti-RBD IgG in COVID-19 patients with severe symptoms were significantly higher than those in patients having mild or moderate symptoms. Other studies have reported that the severe and critical COVID-19 groups had higher levels of anti-SARS-CoV-2 IgG and IgM in comparison to the asymptomatic and mild groups^32,35,36,38^. The mechanism underlying the association of the levels of anti‐SARS-CoV‐2 antibodies with COVID‐19 severity is unclear. Interestingly, COVID-19 patients with more severe symptoms have higher viral loads than those with less severe symptoms, indicating that higher initial viral antigen loads may lead to more potent production of antibodies^39^.

Similar to other serological assays, a primary limitation of our flow cytometric assay is the use of antigens derived from the original SARS-CoV-2 Wuhan strain, a virus that is no longer circulating. The current SARS-CoV-2 variants have undergone numerous mutations, which may have altered their antigenic properties compared to the original strain. As a result, antibodies present in the general population may not be detected with the same sensitivity. Therefore, future studies are necessary to validate the detection of antibodies against the current SARS-CoV-2 variants using our flow cytometric assay.

In summary, our study indicates that the flow cytometric assay, especially using RBD as the antigen, has high sensitivity and specificity, and provides an analytical approach to examine simultaneously anti-SARS-CoV-2 IgG and IgM in humans.

## Supplementary Information

Supplementary Information 1.

Supplementary Information 2.

Supplementary Information 3.

Supplementary Information 4.

Supplementary Information 5.

Supplementary Information 6.

Supplementary Information 7.

Supplementary Information 8.

Supplementary Information 9.

Supplementary Information 10.

## Data Availability

All data generated or analysed during this study are included in this published article and its supplementary information files.
