# Supplementary figures and images for: Flow cytometric analysis of the SARS coronavirus 2 antibodies in human plasma
Source: Sci Rep. 2025 Mar 25;15:10300. doi: 10.1038/s41598-025-92389-8 (PMC11937374; doi:10.1038/s41598-025-92389-8)

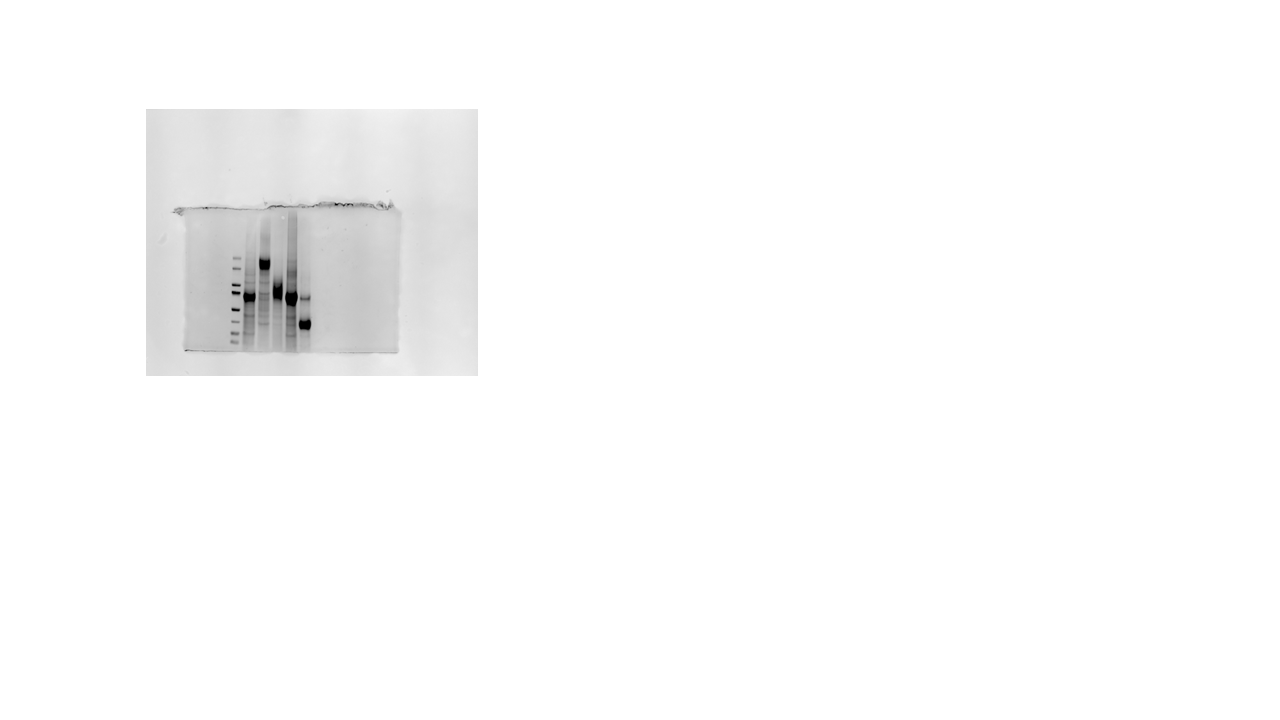

Supplement: Supplementary file 1 — Supplementary Information 1. [file 41598_2025_92389_MOESM1_ESM.tif]

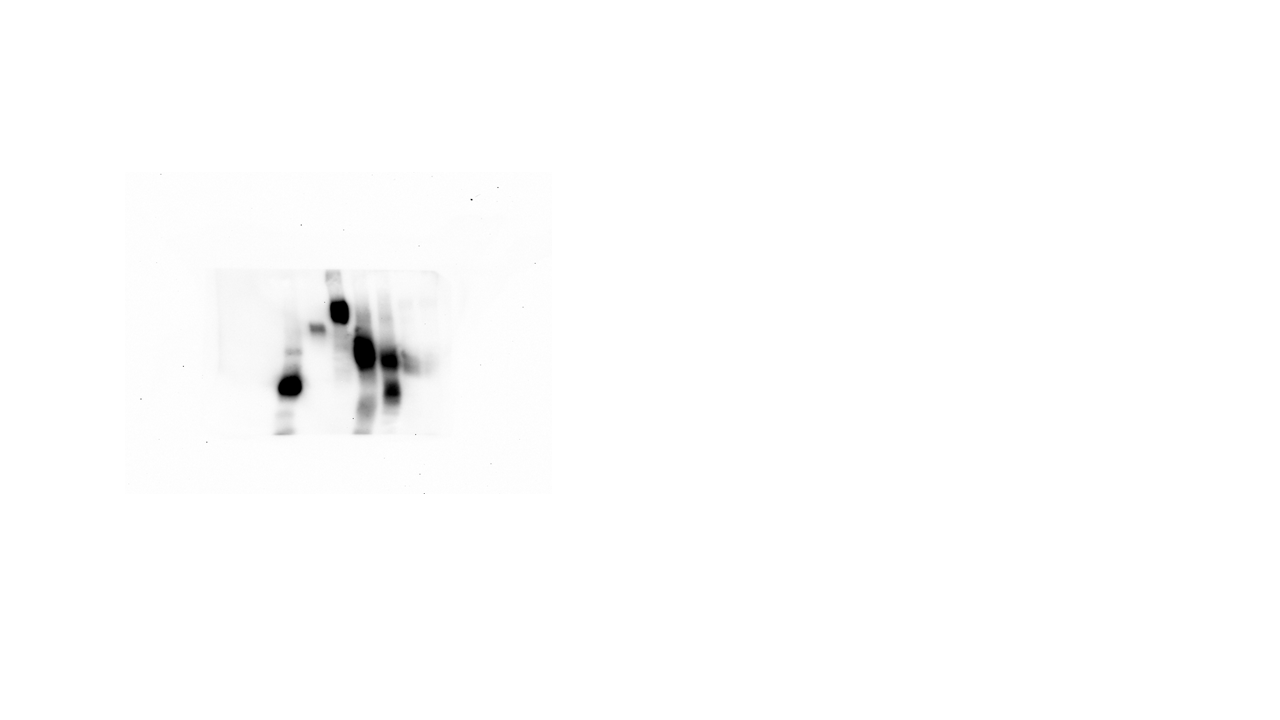

Supplement: Supplementary file 2 — Supplementary Information 2. [file 41598_2025_92389_MOESM2_ESM.tif]

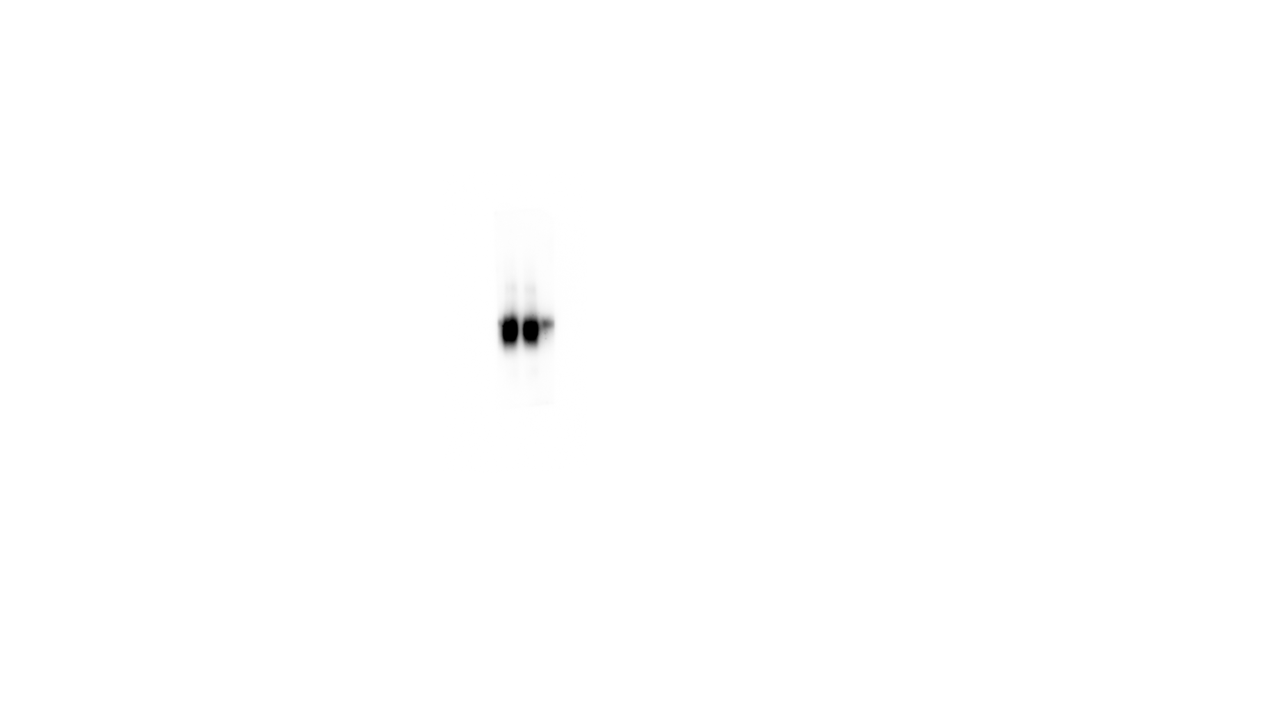

Supplement: Supplementary file 3 — Supplementary Information 3. [file 41598_2025_92389_MOESM3_ESM.tif]

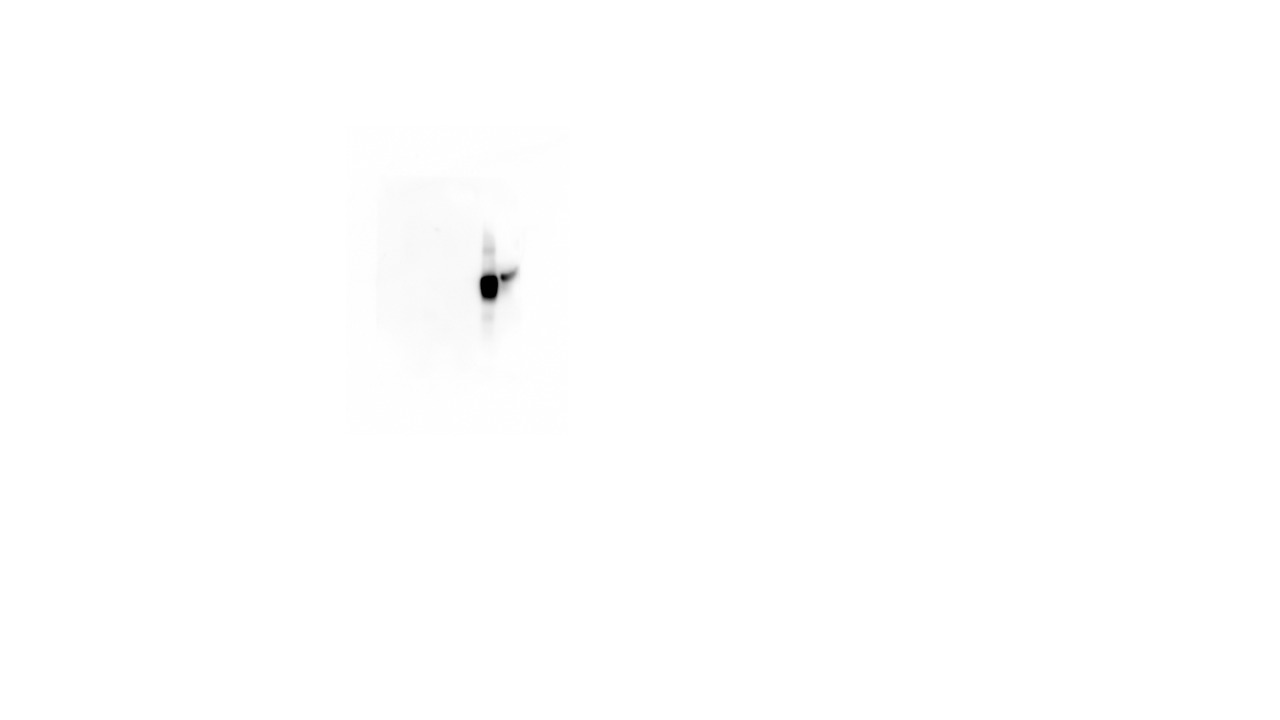

Supplement: Supplementary file 4 — Supplementary Information 4. [file 41598_2025_92389_MOESM4_ESM.tif]

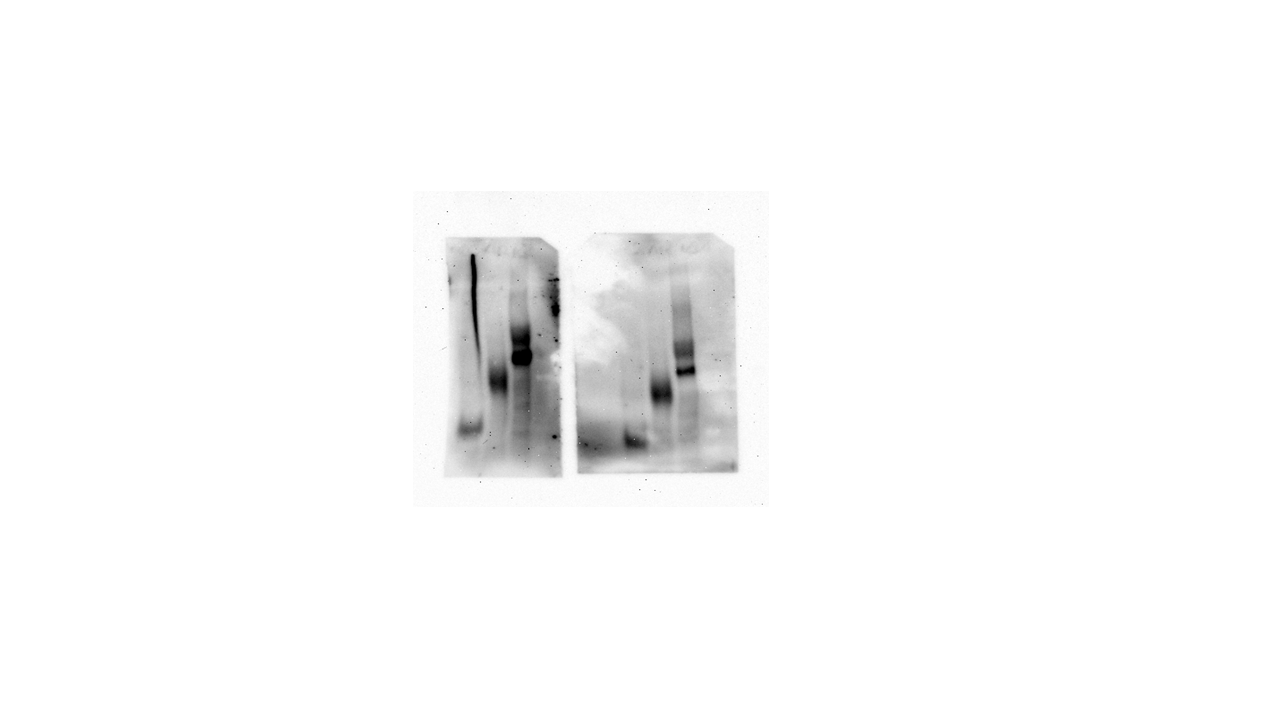

Supplement: Supplementary file 5 — Supplementary Information 5. [file 41598_2025_92389_MOESM5_ESM.tif]

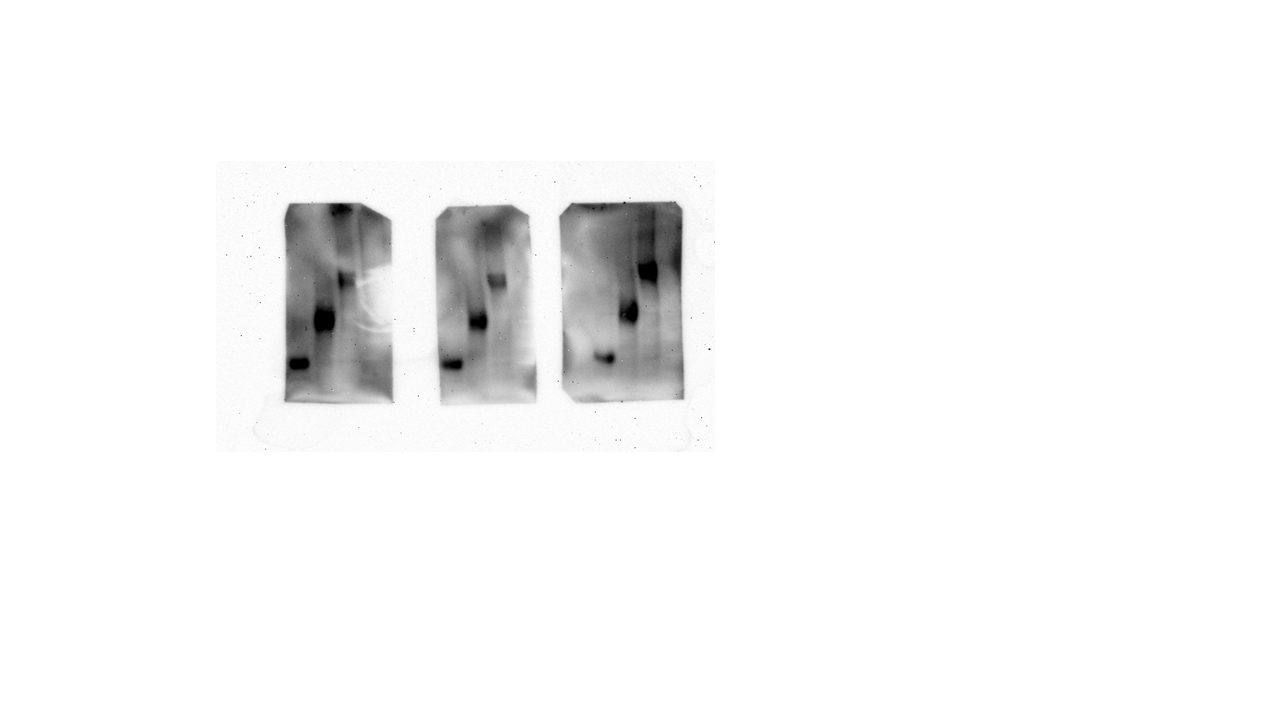

Supplement: Supplementary file 6 — Supplementary Information 6. [file 41598_2025_92389_MOESM6_ESM.tif]

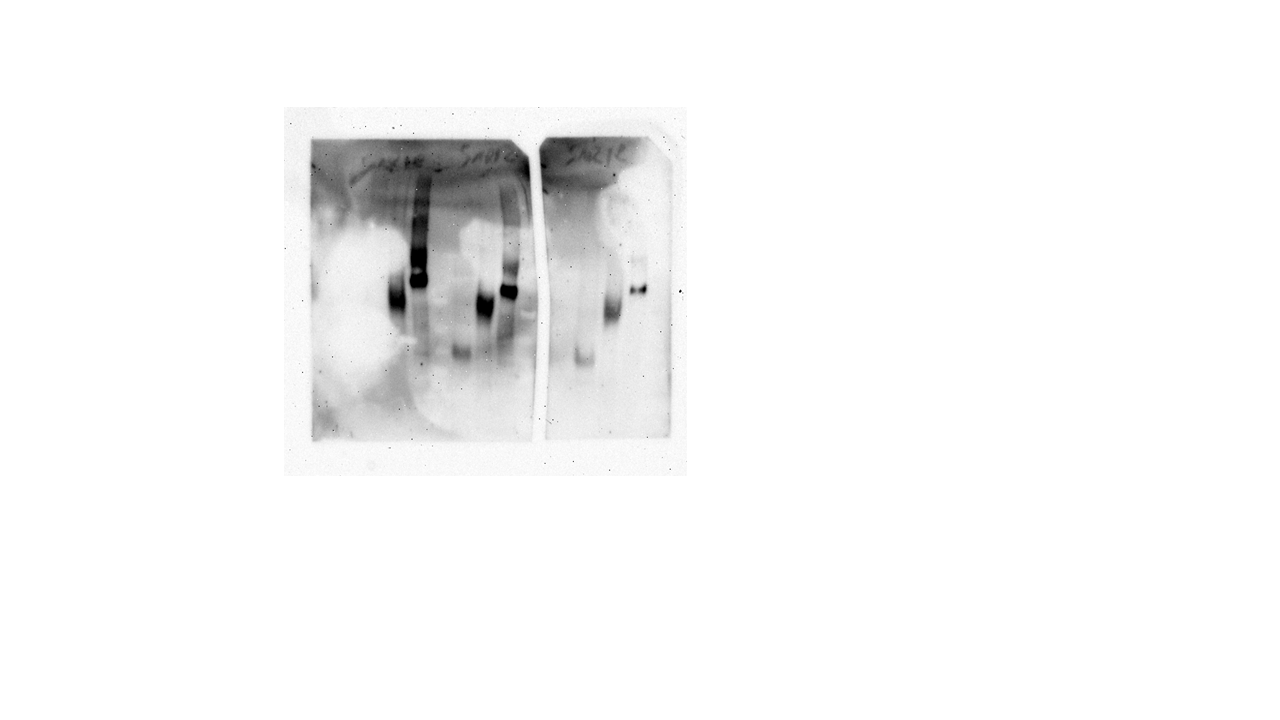

Supplement: Supplementary file 7 — Supplementary Information 7. [file 41598_2025_92389_MOESM7_ESM.tif]

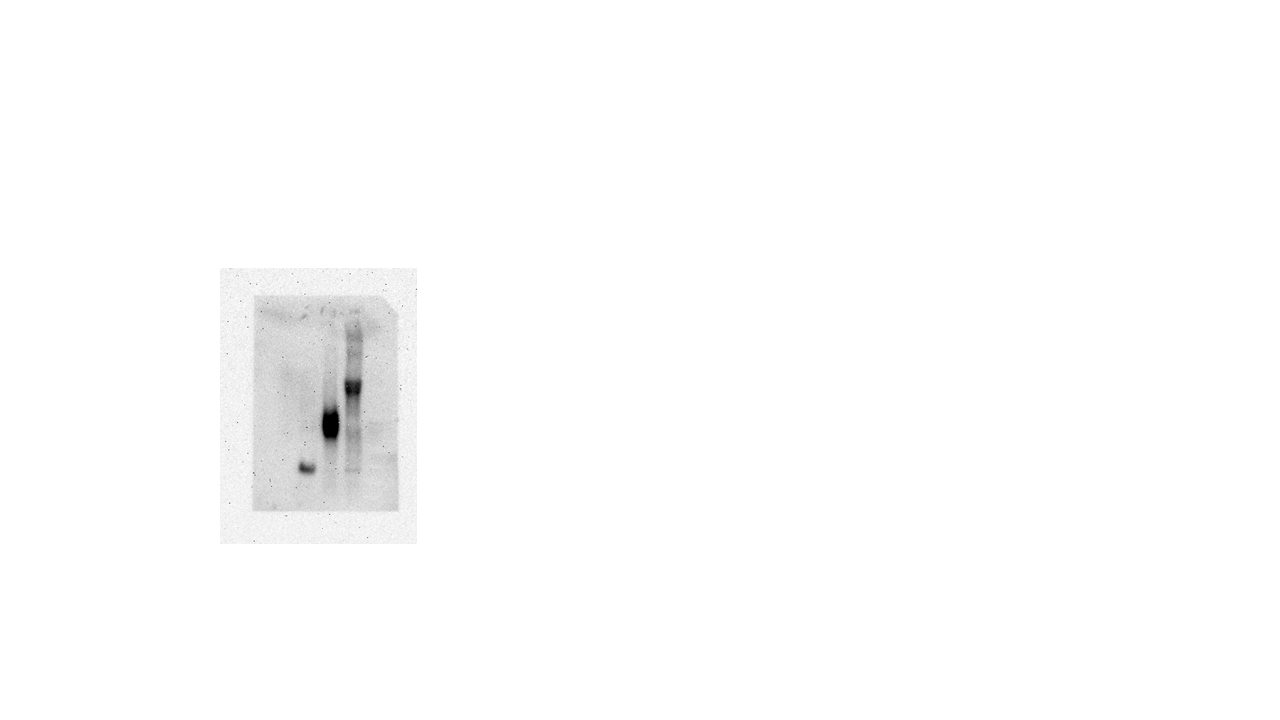

Supplement: Supplementary file 8 — Supplementary Information 8. [file 41598_2025_92389_MOESM8_ESM.tif]

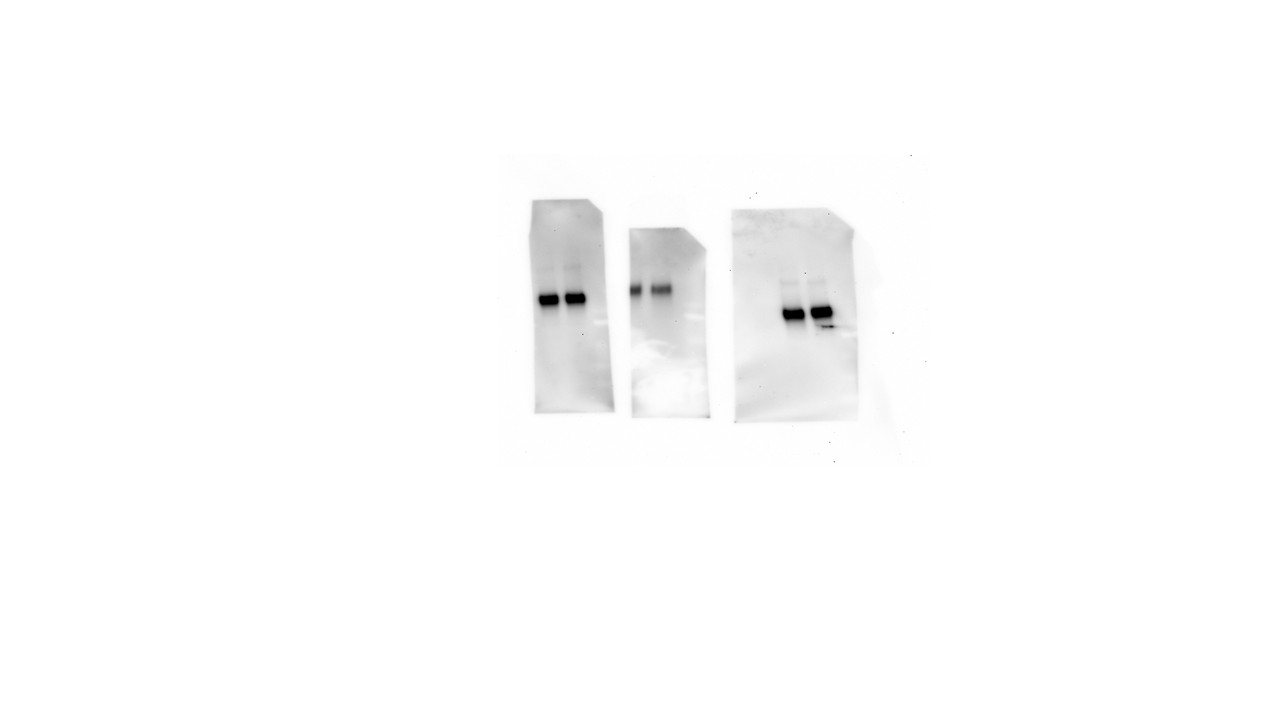

Supplement: Supplementary file 9 — Supplementary Information 9. [file 41598_2025_92389_MOESM9_ESM.tif]
