# Supplementary material for: Flow cytometric analysis of the SARS coronavirus 2 antibodies in human plasma
Source: Sci Rep. 2025 Mar 25;15:10300. doi: 10.1038/s41598-025-92389-8 (PMC11937374; doi:10.1038/s41598-025-92389-8)
Supplement: Supplementary file 10 — Supplementary Information 10. [file 41598_2025_92389_MOESM10_ESM.docx]

Supplementary Table 1. Prevalence of anti-SARS-CoV-2 IgG or IgM in the plasma of healthy blood donors and COVID-19 patients^a^

| Proteins | Healthy blood donors | | | COVID-19 patients | | |
| --- | --- | --- | --- | --- | --- | --- |
|  | IgG only | IgM only | IgG & IgM | IgG only | IgM only | IgG & IgM |
| S1 | 1.0% (1)^b^ | 0.0% (0) | 0.0% (0) | 54.0% (54) | 0.0% (0) | 43.0% (43) |
| S1A | 0.0% (0) | 0.0% (0) | 0.0% (0) | 72.0% (72) | 0.0% (0) | 0.0% (0) |
| RBD | 0.0% (0) | 1.0% (1) | 0.0% (0) | 19.0% (19) | 0.0% (0) | 81.0% (81) |
| S2 | 1.0% (1) | 0.0% (0) | 0.0% (0) | 40.0% (40) | 6.0% (6) | 3.0% (3) |
| N | 1.0% (1) | 1.0% (1) | 0.0% (0) | 69.0% (69) | 0.0% (0) | 31.0% (31) |

^a^ Plasma samples of healthy blood donors (n = 100) and COVID-19 patients (n = 100) were analyzed using flow cytometry.

^b^ Number in parenthesis indicate the number of samples with an anti-SARS-CoV-2 antibody response above the cutoff-point.

Supplementary Table 2. Prevalence of anti-SARS-CoV-2 IgG or IgM in COVID-19 patients by sex

| Proteins | IgG | | IgM | | |  |
| --- | --- | --- | --- | --- | --- | --- |
|  | Male | Female | | Male | Female | |
| S1 | 95.1% (39/41) | 98.3% (58/59) | | 51.2% (21/41) | 37.3% (22/59) | |
| S1A | 75.6% (31/41) | 69.5% (41/59) | | 0% (0/41) | 0% (0/59) | |
| RBD | 100% (41/41) | 100% (59/59) | | 85.3% (35/41) | 78.0% (46/59) | |
| S2 | 58.5% (24/41) | 32.2%** (19/59) | | 2.4% (1/41) | 13.6% (8/59) | |
| N | 100% (41/41) | 100% (59/59) | | 31.7% (13/41) | 30.5% (18/59) | |

**Significant (p *<* 0.01) difference between males and females.

Supplementary Table 3. Prevalence of anti-SARS-CoV-2 IgG or IgM in COVID-19 patients by age

| Proteins | IgG | | | | IgM | | | |
| --- | --- | --- | --- | --- | --- | --- | --- | --- |
|  | ≤ 29 Yrs | 30 – 49 Yrs | 50 – 64 Yrs | ≥ 65 Yrs | ≤ 29 Yrs | 30 – 49 Yrs | 50 – 64 Yrs | ≥ 65 Yrs |
| S1 | 100% (16/16) | 94.9% (37/39) | 97.3% (36/37) | 100% (8/8) | 43.8% (7/16) | 33.3% (13/39) | 48.6% (18/37) | 62.5% (5/8) |
| S1A | 81.3% (13/16) | 74.4% (29/39) | 64.9% (24/37) | 75.0% (6/8) | 0% (0/16) | 0% (0/39) | 0% (0/37) | 0% (0/8) |
| RBD | 100% (16/16) | 100% (39/39) | 100% (37/37) | 100% (8/8) | 93.8% (15/16) | 76.9% (30/39) | 78.4% (29/37) | 87.5% (7/8) |
| S2 | 43.8 (7/16) | 35.9% (14/39) | 45.9% (17/37) | 62.5% (5/8) | 12.5% (2/16) | 5.1% (2/39) | 13.5% (5/37) | 0% (0/8) |
| N | 100% (16/16) | 100% (39/39) | 100% (37/37) | 100% (8/8) | 18.8% (3/16) | 30.8% (12/39) | 32.4% (12/37) | 50.0% (4/8) |

Supplementary Table 4. Prevalence of anti-SARS-CoV-2 IgG or IgM in COVID-19 patients by race^a^

| Proteins | IgG | | IgM | | |  |
| --- | --- | --- | --- | --- | --- | --- |
|  | Caucasian | Hispanic | | Caucasian | Hispanic | |
| S1 | 100% (19/19) | 95.5% (64/67) | | 36.8% (7/19) | 41.8% (28/67) | |
| S1A | 57.9% (11/19) | 73.1% (49/67) | | 0% (0/19) | 0% (0/67) | |
| RBD | 100% (19/19) | 100% (67/67) | | 78.9% (15/19) | 80.6% (54/67) | |
| S2 | 31.6% (6/19) | 41.8% (28/67) | | 10.5% (2/19) | 6.0% (4/67) | |
| N | 100% (19/19) | 100% (67/67) | | 21.1% (4/19) | 34.3% (23/67) | |

^a^Due to limited number of samples from Asian, black, and unknown (Table 1), they were not included in the analysis.

Supplementary Table 5. Prevalence of anti-SARS-CoV-2 IgG or IgM in COVID-19 patients by days after the onset of symptoms

| Proteins | IgG | | | IgM | | |
| --- | --- | --- | --- | --- | --- | --- |
|  | 0 – 6 days | 7 – 14 days | ≥ 14 days | 0 – 6 days | 7 – 14 days | ≥ 14 days |
| S1 | 97.4% (37/38) | 94.4% (17/18) | 100% (12/12) | 39.5% (15/38) | 44.4% (8/18) | 50.0% (6/12) |
| S1A | 73.7% (28/38) | 72.2% (13/18) | 83.3% (10/12) | 0% (0/38) | 0% (0/18) | 0% (0/12) |
| RBD | 100% (38/38) | 100% (18/18) | 100% (12/12) | 76.3% (29/38) | 83.3% (15/18) | 83.3% (10/12) |
| S2 | 57.9% (22/38) | 44.4% (8/18) | 25.0% (3/12) | 7.9% (3/38) | 11.1% (2/18) | 0% (0/12) |
| N | 100% (38/38) | 100% (18/18) | 100% (12/12) | 28.9% (11/38) | 38.9% (7/18) | 25.0% (3/12) |

Supplementary Table 6. Prevalence of anti-SARS-CoV-2 IgG or IgM in COVID-19 patients with severity of symptoms

| Proteins | IgG | | | | IgM | | | | | | | | | | |
| --- | --- | --- | --- | --- | --- | --- | --- | --- | --- | --- | --- | --- | --- | --- | --- |
|  | Asymptotic | Mild | Moderate | Severe | | Asymptotic | | Mild | | Moderate | | Severe | |  |  |
| S1 | 100% (12/12) | 92.7% (38/41) | 100% (19/19) | 100% (7/7) | | | 66.7% (8/12) | | 46.3% (19/41) | | 42.1% (8/19) | | 85.7% (6/7) | |  |
| S1A | 83.3% (10/12) | 65.9% (27/41) | 73.7% (14/19) | 85.7% (6/7) | | | 0% (0/12) | | 0% (0/41) | | 0% (0/19) | | 0% (0/7) | |  |
| RBD | 100% (12/12) | 100% (41/41) | 100% (19/19) | 100% (7/7) | | | 91.7% (11/12) | | 80.5% (33/41) | | 89.5% (17/19) | | 100% (7/7) | |  |
| S2 | 58.3% (7/12) | 31.7% (13/41) | 42.1% (8/19) | 85.7% (6/7) | | | 16.7% (2/12) | | 7.3% (3/41) | | 5.3% (1/19) | | 28.6% (2/7) | |  |
| N | 100% (12/12) | 100% (41/41) | 100% (19/19) | 100% (7/7) | | | 58.3% (7/12) | | 36.6% (15/41) | | 21.1% (4/19) | | 42.9% (3/7) | |  |
